# Inflammatory stimuli alter bone marrow composition and compromise bone health in the malnourished host

**DOI:** 10.3389/fimmu.2022.846246

**Published:** 2022-08-02

**Authors:** E. Yaneth Osorio, Zbigniew Gugala, Grace T. Patterson, Genesis Palacios, Erika Cordova, Ashanti Uscanga-Palomeque, Bruno L. Travi, Peter C. Melby

**Affiliations:** ^1^ Department of Internal Medicine, Division of Infectious Diseases, University of Texas Medical Branch, Galveston, TX, United States; ^2^ Department of Orthopedic Surgery and Rehabilitation, The University of Texas Medical Branch, Galveston, TX, United States; ^3^ Department of Parasitology, Universidad de la Laguna, San Cristóbal de La Laguna, Spain; ^4^ Department of Microbiology and Immunology, University of Texas Medical Branch, Galveston, TX, United States; ^5^ Center for Tropical Diseases and Institute for Human Infection and Immunity, University of Texas Medical Branch, Galveston, TX, United States; ^6^ Department of Pathology, University of Texas Medical Branch, Galveston, TX, United States

**Keywords:** malnutrition, bone marrow, bone, adipocyte, osteoclast, lipopolysaccharide, *Leishmania donovani*

## Abstract

Inflammation has a role in the pathogenesis of childhood malnutrition. We investigated the effect of malnutrition and inflammatory challenge on bone marrow composition and bone health. We studied an established murine model of moderate acute malnutrition at baseline and after acute inflammatory challenge with bacterial lipopolysaccharide (LPS), a surrogate of Gram-negative bacterial sepsis, or *Leishmania donovani*, the cause of visceral leishmaniasis. Both of these infections cause significant morbidity and mortality in malnourished children. Of the 2 stimuli, LPS caused more pronounced bone marrow changes that were amplified in malnourished mice. LPS challenge led to increased inflammatory cytokine expression (*Il1b*, *Il6*, and *Tnf*), inflammasome activation, and inflammatory monocyte accumulation in the bone marrow of malnourished mice. Depletion of inflammatory monocytes in *Csfr1*-LysMcre-DT malnourished mice significantly reduced the inflammasome activation and IL1-ß production after LPS challenge. The inflammatory challenge also led to increased expansion of mesenchymal stem cells (MSCs), bone marrow adiposity, and expression of genes (*Pparg*, *Adipoq*, and *Srbp1*) associated with adipogenesis in malnourished mice. This suggests that inflammatory challenge promotes differentiation of BM MSCs toward the adipocyte lineage rather than toward bone-forming osteoblasts in the malnourished host. Concurrent with this reduced osteoblastic potential there was an increase in bone-resorbing osteoclasts, enhanced osteoclast activity, upregulation of inflammatory genes, and IL-1B involved in osteoclast differentiation and activation. The resulting weakened bone formation and increased bone resorption would contribute to the bone fragility associated with malnutrition. Lastly, we evaluated the effect of replacing lipid rich in omega-6 fatty acids (corn oil) with lipid-rich in omega-3 fatty acids (fish oil) in the nutrient-deficient diet. LPS-challenged malnourished mice that received dietary fish oil showed decreased expression of inflammatory cytokines and *Rankl* and reduced osteoclast differentiation and activation in the bone marrow. This work demonstrates that the negative effect of inflammatory challenge on bone marrow is amplified in the malnourished host. Increasing dietary intake of omega-3 fatty acids may be a means to reduce inflammation and improve bone health in malnourished children.

## Introduction

Human malnutrition is a complex disorder with deficiencies of micronutrients often superimposed on deficits of protein and energy due to an inadequate diet (1, 2). In 2020, about 149.2 million children (22% of children globally) under five years of age suffered from stunting (chronic malnutrition) and 45.4 million (6.7% of children globally) had wasting, which is made up of moderate (MAM) and severe acute malnutrition (SAM) (3). The combination of stunting, severe wasting, and pre-natal growth restriction cause about 2·2 million deaths and 21% of disability-adjusted life-years (DALYs) for children under 5 years old (4). In general, the incidence of MAM is about twice that of SAM. Children with SAM have about a 9-fold increase in mortality whereas it is increased 3-fold for children with MAM (4, 5). In acute childhood malnutrition, loss of body mass is relatively greater than the loss of linear growth so that the weight-for-height z score is between -2 and -3 for moderate acute malnutrition (MAM) and less than -3 for severe acute malnutrition. Chronic malnutrition tends to impact long-term linear growth so that the height-for-age z score is between -2 and -3 for moderate and less than -3 for severe stunting.

The pathophysiology of malnutrition is incompletely understood (6). Environmental, social, demographic, and economic conditions impact the availability of dietary nutrients crucial for the growth, development, and maintenance of body homeostasis. Inadequate energy intake leads to multiple pathophysiologic adaptations including restriction of musculoskeletal growth, loss of body fat, depletion of glycogen stores, and reduced energy expenditure. Metabolic and hormonal changes lead to lipolysis and oxidation of free fatty acids, and the breakdown of muscle into amino acids that can be converted into glucose through gluconeogenesis (7, 8). This metabolic disruption is worsened by inflammatory mediators, especially tumor necrosis factor (TNF) and interleukin-1 beta (IL-1β), which suppress the appetite and increase catabolism of adipose tissue and muscle. Consequences in the short-term include growth faltering and susceptibility to infection, and in the long-term impaired psychomotor and cognitive development (9).

There is a growing body of evidence that changes in the intestinal microbiota and intestinal and systemic inflammation, with or without overt bacterial infection, underpin pathophysiological processes in the malnourished host. Recently, we demonstrated an increase in basal inflammatory cytokines, impaired intestinal barrier function, circulating endotoxin, and an exaggerated response to inflammatory stimuli in children with MAM (10). Similar features are reported in our studies in a mouse model that mimics the features of MAM (11). These features were associated with the development of a proinflammatory intestinal microbiota.

Malnutrition is a risk factor for increased severity of infectious diseases, most prominently diarrhea, lower respiratory tract infection, and bacterial sepsis. The mechanisms responsible for impaired host defense in childhood malnutrition are not fully understood. One source of susceptibility to infections linked with nutritional deficiencies is the disruption of enteric barrier function that promotes the systemic translocation of bacteria and bacterial products such as LPS (9, 10). The metabolic cost of the activated immune response and the consequent intestinal or systemic inflammation during the infection support the vicious cycle of nutritional deficiency and impaired host defense. Pre-existing nutritional deficiencies enhance the malnourished host’s susceptibility to respiratory diseases such as influenza, pneumonia, and tuberculosis, as well as those caused by protozoal pathogens such as *Giardia*, *Cryptosporidium*, *Plasmodium*, *Leishmania*, and helminths [reviewed in (12)].

The impact of systemic infections on bone health in childhood malnutrition is evident in stunting. Malnutrition increases by 37% the risk of diarrhea, and five or more episodes of diarrhea before 24 months of age increase the risk of stunting by 25% (13). HIV infection and antiretroviral therapy are associated with decreased bone strength, low mineral density, and low bone mass which increase fracture risk in adulthood (14, 15). Malnourished children have a 12-fold increase in susceptibility to visceral leishmaniasis (16). More than half of the pediatric VL patients had vitamin D deficiency (17), which affects both skeletal growth and bone mineral density (18).

The bone marrow (BM) and lymphoid organs are particularly vulnerable to the effects of nutrient deficiencies because of the high rate of cell proliferation and self-renewal. Findings in experimental models of protein deficiency show reduced cellularity in bone marrow, spleen, and lymph nodes (12). Bone marrow stroma of mice fed a protein-deficient diet fails to sustain hematopoietic stem cells and progenitors (19), in part because of cell cycle arrest (20, 21). Mesenchymal stem cells preferentially differentiate into adipose cells in protein-deficient mice, leading to an altered cytokine microenvironment and compromised hematopoiesis (19). Studies of adults experiencing starvation demonstrated commitment of mesenchymal stem cell differentiation to fat cells that resulted in reduced differentiation of bone-forming osteoblasts, causing pathological conditions, such as osteopenia, osteoporosis, and malnutrition-associated bone fractures (20, 21).

Innate immune cells, such as monocytes, macrophages, and dendritic cells (DCs) originate from common myeloid precursors in the BM (22, 23). The same common myeloid progenitor gives rise to osteoclasts, which play a primary role in bone resorption. Osteoclast differentiation from myeloid precursors is regulated by the receptor activator of nuclear factor κB ligand (RANKL), which is induced by TNF (24, 25). Conversely, osteoprotegerin (OPG), which is a soluble decoy receptor of RANKL, inhibits osteoclast activation (26). Alterations of the delicate balance between RANKL and OPG result in elevated bone resorption over bone formation, which is characteristic of inflammation-associated bone diseases, such as osteopenia, osteoporosis, arthritis, and bone lysis in periodontal diseases (27). Factors other than inflammatory cytokines, such as bacterial products, endotoxin, prostaglandin E_2,_ and the inflammasome can also exert direct and indirect effects on osteoclast activation and differentiation (28–30). The relationship between osteoclasts and myeloid cells goes beyond having a common progenitor as monocytes, macrophages, and DCs can differentiate into multinucleated osteoclasts under conditions of pathological inflammation (31–35). This underscores the versatility of myeloid cells and their multiple roles in the bone marrow compartment and bone health.

To our knowledge, there have been no reports of the effect of malnutrition and inflammation on bone marrow and bone health in children. In the elderly population, chronic inflammatory diseases aggravate the effects of malnutrition leading to frailty, sarcopenia, and falls, thereby increasing the incidence of bone fractures (36). Therefore, we used an established mouse model of MAM (37–41) to investigate the impact of an acute infectious or inflammatory challenge on bone marrow and bone health. In this model, malnutrition led to systemic inflammation, impaired intestinal barrier function, circulating endotoxin, and an exaggerated response to inflammatory stimuli that promoted weight loss (11). Here we show that malnutrition also conditions the bone marrow for an altered response to 2 different inflammatory stimuli. We used challenge with bacterial LPS as a surrogate of Gram-negative bacterial sepsis (42) (e.g. *Salmonella* and *E. coli* bacteremia) because of its significant morbidity and mortality in children with SAM (43–45). We also used challenge with the protozoan parasite *Leishmania donovani*, the cause of visceral leishmaniasis (VL), because malnutrition is a major risk factor for VL (46–48), and our prior studies demonstrated increased early susceptibility of malnourished mice to challenge with this pathogen (38, 40). Our results show that after the inflammatory challenge, the bone marrow of malnourished mice compared to controls had increased inflammatory cytokine expression and inflammatory monocyte accumulation, greater expansion of mesenchymal stem cells (MSCs) and bone marrow adiposity, and enhanced osteoclast differentiation and activation. These changes were associated with differential expression of transcription factors that regulate differentiation of bone marrow adipocytes and osteoclasts. Replacing omega-6-rich lipids with omega-3-rich lipids in the nutrient-deficient diet ameliorated malnutrition-related bone marrow inflammation and osteoclast differentiation and activation.

## Materials and methods

### Mice

Age-matched, 3-week-old female Balb/c weanling mice were purchased from Envigo (Houston, Texas). Malnourished mice were fed for 28 days with a polynutrient deficient diet, containing 3% protein, low zinc, and iron (TD.99075, Envigo), and the control group (well-nourished) was fed with a nutrient-sufficient diet of 16.9% protein, normal zinc, and iron (TD.99103, Envigo) as described (38, 40). Lipid in the two mouse chows was supplied by corn oil (9%) and the diets were made isocaloric by varying the carbohydrate content. Mice were pair-fed with the malnourished mice being provided 90% (by weight) of the food consumed by the control group. For studies of the effect of dietary fish oil, the 9% corn oil in the polynutrient deficient diet was replaced with 9% fish oil (TD.160285, Envigo).

### Mice challenge

After 28 days on the control or nutrient-deficient diet, mice were challenged with LPS (4mg/kg intraperitoneally) from *Escherichia Coli* O111B4 (Sigma) as a model of Gram-negative bacterial sepsis (42) and evaluated 24h later. Mice challenged with *Leishmania donovani* were infected intradermally (ID) in the dorsum of the foot with 10^6^ purified *L. donovani* metacyclic promastigotes and evaluated 72h later as described (40). To study the effect of inflammatory monocytes, we used a Cre-inducible diphtheria toxin (DT) expression model (49). In brief, 6–8-week-old female and male offspring were obtained from B6.129P2-Lyz2tm1(cre)Ifo/J (Jackson stock # 004781) crossed with C57BL/6-Tg (Csf1r-HBEGF/mCherry)1Mnz/J (Jackson stock #024046). Offspring carrying the *Csfr1*-LysMcre-DT phenotype and controls were placed on the experimental diet for 28 days. To deplete inflammatory monocytes, mice were given 5 ng of DT (Sigma) intraperitoneally, per gram body weight every 24h.

### Microscopy

Mouse sternums were snap-frozen in cryomolds with OCT using an isopentane bath. Sternums were stored at -80C until analysis. Longitudinal cryosections (6 µm) were obtained in a Leica cryostat and were stained with lipid stain solution Oil-red O (Abcam) according to manufacturer instructions. After Oil-red O staining, slides were double-stained with alkaline phosphatase ALP (Takara) to differentiate osteoblasts. Osteocytes were identified by location in the trabecular bone, characteristic stellate cell morphology, and the expected expression of alkaline phosphatase. To identify osteoclasts, slides were stained for Tartrate-resistant acid phosphatase (TRAP) (TRACP kit, Takara). Osteoclasts were further identified as multinucleated cells following the manufacturer’s instructions. Images were captured with 16X Apochromat NIR objective lenses in a Nikon microscope (Elipse 80i) with a NIS-elements BR™ Software. Images, of at least 20 fields per group, were further analyzed with the software Image Processing and Analysis in Java Software (ImageJ) and QuPath-02.3. Image analysis in Image J was done according to software instructions. In brief, TIF color images were uploaded and converted to 8-bit. The background was subtracted and the pixel value was calibrated to length in microns and measurements were set (area). The threshold of the positive signal was adjusted and applied globally to all images to compare. The percentage of the positive area was determined in the images covering the total length of each sternum. The number of positive cells was determined with the tool for particle analysis (size adjusted 0-1). Image analysis in QuPath was done as described (50).

### Flow cytometry

Bone marrow cells were obtained from the femurs of mice. Femurs were carefully broken in a ceramic grinder and treated for 10 min with Accutase solution (Millipore) to release bone marrow cells. After collecting and washing the cells, they were adjusted to 10^6^ per tube. Cells were blocked with 0.5 µg of Fc block and stained with a viability marker. Cells were identified with the following markers: inflammatory monocytes: CD11b+, CD11c-, Ly6G-Ly6C^int/hi+^ (51); T cell inducers of osteoclasts: CD3+RANKL+; osteoclast precursors: CD11b^-/low^ CD45+ckit+(CD117+) CD115+ (52); mesenchymal stem cells: CD45-CD29+CD105+ (R&D, Mesenchymal stem cell markers) (**Table S1**). After staining, cells were washed and treated with a 1-step fix/lyse solution (eBioscience). Intracellular cytokines were determined after 2-4 h of incubation of 10^6^ bone marrow cells with 1µg/mL of Golgi stop (BD). Fixed cells were acquired with a benchtop Stratedigm flow cytometer (S500) or BD LRSFortessa flow cytometer (BD Biosciences) and analyzed with FlowJo (V10). Panels were designed to minimize overlapping fluorescence between antibodies with FluoroFinder (Panel Builder 3.0) online platform. Thresholds for gating were established with FMO controls. The gating strategy that was followed is shown in **Supplementary Figure 1A–E**.

### Gene expression and inflammasome assay

RNA from bone marrow cells was isolated with the RNeasy kit (Qiagen) and treated with DNase I (Turbo DNase, Ambion) followed by reverse transcription (High-capacity Reverse transcription kit, Thermofisher Sci.). Primers to amplify target genes were selected from the PrimerBank database (Wang, Spandidos et al., 2012) (**Table S2**). Targets were amplified by real-time PCR (qPCR) and detected with SYBR Green dye (Bio-Rad). The fold change of gene expression was calculated with the delta CT method and 18S as the reference gene. The fold-change was calculated relative to the value in uninfected naïve mice as specified in each figure legend. Inflammasome activation was assessed with Caspase Glo-1 inflammasome kit (Promega) according to manufacturer instructions. Bone marrow cells from *Csfr1*-LysMcre-DT mice, selected as above, were plated in 96-well white luminometry plates (50,000 cells/well) and were exposed *in vitro* for 1h to LPS or Leishmania antigens (Freeze/thaw parasites) for luminometry reading.

### Osteoclast differentiation and *ex vivo* cultures

Bone marrow cells were cultured in medium containing high glucose DMEM with 10% heat-inactivated fetal bovine serum (FBS), 1 mM sodium pyruvate (Gibco), 1X MEM amino acids solution (Sigma), 10 mM HEPES buffer (Cellgro) and 100 IU/mL Penicillin/100mg/mL Streptomycin (Gibco). Cells were added to 24-well plates containing round glass coverslips at a concentration of 0.5 x 10^6^ cells per well in 300 µL medium. To differentiate osteoclasts, 40 ng/mL RANKL and 20 ng/mL macrophage colony-stimulating factor (M-CSF) (Biolegend) were added to the culture media. The culture medium was changed at 72h of culture and cells were evaluated after 7 days of differentiation at 37°C, 5% CO_2_. Cells were fixed and stained with TRAP as above. Multinuclear mature osteoclasts were identified by nuclear staining with Methyl green (TRACP kit, Takara) or 7AAD and were evaluated by microscopy or flow cytometry respectively (53). For some *ex vivo* cultures the following were added: 100ng/mL DHA (Docosahexaenoic acid (Sigma) diluted in fatty acid-free Bovine Serum Albumin (BSA) at 10:1 ratio; 10ng/mL LPS; 6mg/mL homogenates from inguinal white fat adipose tissue; *Leishmania donovani* antigen (1:1 thaw-frozen promastigotes), or vehicle diluent for control wells.

### Statistical analysis

Data were analyzed with GraphPad Prism 9.0, Software (Graphpad, LLC) according to software recommendations. Comparisons between 2 groups were evaluated with two tail Mann-Whitney U test for non-parametric data or two tail unpaired t test for normally distributed data. Comparisons between more than 2 groups were evaluated with Kruskal-Wallis for non-parametric data or ANOVA for normally distributed with *post hoc* correction for multiple comparisons (Bonferroni or Tukey). Differences in proportions were assessed with the Chi-squared test. Data from histological slides were collected from at least 20 randomly selected microscopic fields. *In vitro* experiments were performed with at least four replicates per condition. *In vivo* experiments were performed with 4-5 mice per group. Experiments were repeated at least twice.

## Results

### Malnourished mice have reduced body weight and length and reduced bone length

**Figure 1:**
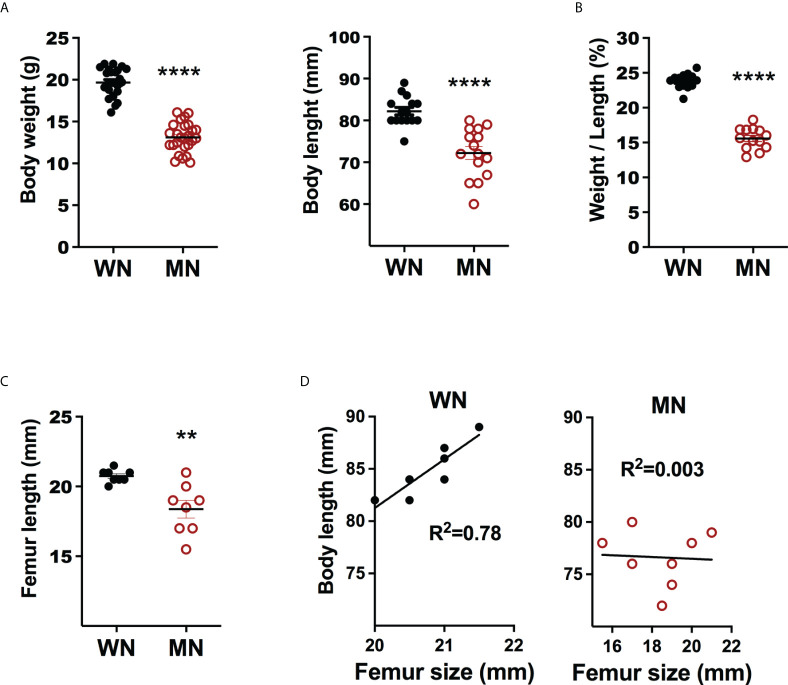
Body and bone metrics of control and malnourished mice. 3-week old BALB/c mice were placed on the isocaloric control and nutrient-deficient diets for 28 days and body weight and body and femur length measured. **(A)** Body weight (g) and body length (mm) of naïve control (well-nourished; WM) and malnourished (MN) mice. Data pooled of 4 different experiments with >5 mice per group in each experiment. **(B)** Ratio of body weight (g) to body length (mm) (W/L) in naive well-nourished (WN) and malnourished mice (MN). Data pooled of 2 experiments with 8 mice per group. **(C)** Femur length (mm) in WN and MN mice. Data are from a single experiment representative of 2 experiments with 8 mice per group, **(D)** Correlation of body length vs. femur length in WN and MN mice. Data are from a single experiment representative of 2 experiments with 8 mice per group. **p<0.01, ****p<0.0001.

We evaluated the effect of moderate acute malnutrition (diet deficient in protein, iron, and zinc) on body weight and length, and bone length. As we demonstrated previously, the malnourished mice had a flat growth curve so that when the control mice reached a mature weight 28 days after weaning to a normal diet, the malnourished (MN) mice had approximately 30% less body weight and a 15% reduction in body length (**Figure 1A**). The disproportionately reduced weight over length (**Figure 1B**) is characteristic of acute malnutrition. Malnourished mice also showed significant reductions in the length of the femur (**Figure 1C**). Body length and femur length were strongly correlated in control mice but not in malnourished mice (**Figure 1D**). This suggests that bone health may be uniquely impacted by acute malnutrition.

### Bone marrow and bone adiposity is enhanced after acute inflammatory challenge of malnourished mice

**Figure 2:**
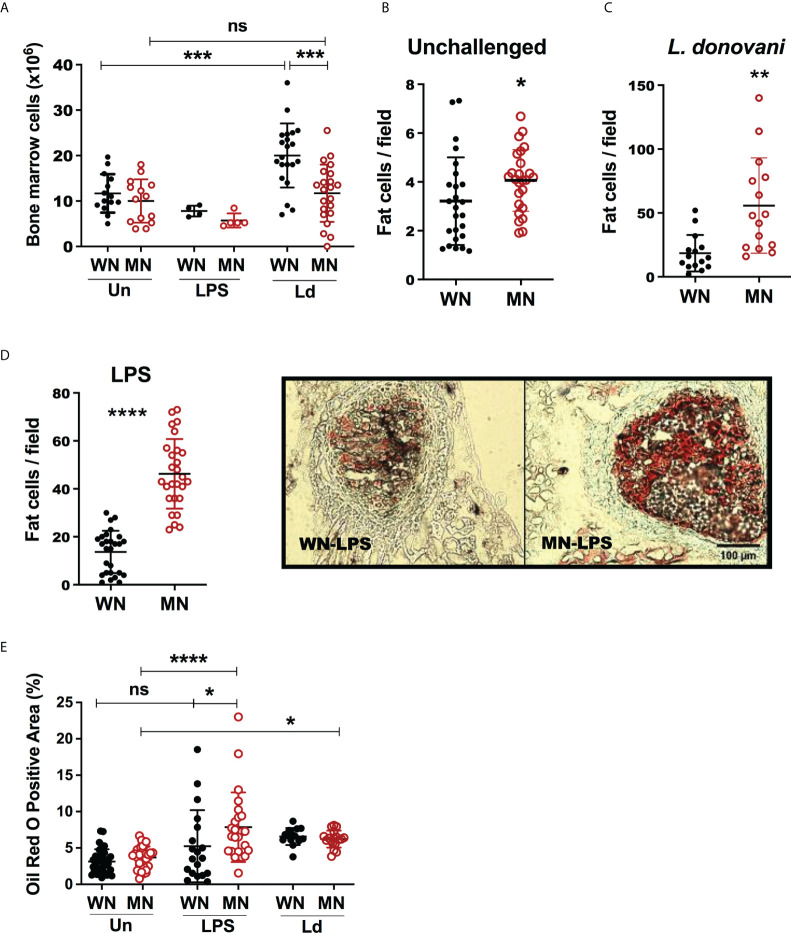
Malnutrition leads to increased bone marrow adiposity. Well-nourished (WN) or malnourished (MN) mice were challenged with lipopolysaccharide (LPS) or *L. donovani* (*Ld*) and compared with naïve unchallenged (Un) mice. *L. donovani*-challenged mice were evaluated at 72h post-challenge and LPS-challenged mice were evaluated at 24h post-challenge. **(A)** Number of cells in bone marrow from femur enumerated by microscopy. **(B)** Number of lipid-filled Oil-Red O positive (Oil-red O+) cells in sternum cryosections from uninfected mice. **(C)** Number of cells Oil-Red O positive (Oil-red O+) in sternum cryosections from *L. donovani* challenged mice at 72h post-challenge. **(D)** Left panel: Number of Oil-Red O+ bone marrow cells per field in sternum cryosections from LPS-challenged mice. Right panel: image showing Oil-red O+ cells in hematopoietic centers observed in sternum cryosections from LPS challenged mice. **(E)** Oil-Red O+ area (hematopoietic centers + bone areas) in sternum cryosections. N=5 mice per group, 2 experiments. *p<0.05, **p<0.01, ***p<0.001, ****p<0.0001. ns, not significant.

We evaluated the effect of inflammatory or infectious challenges on bone marrow cellularity in malnourished mice. Despite the femur length of the malnourished mice being smaller in the basal state, there was only a slight reduction in the total number of bone marrow cells recovered from malnourished and control mice (**Figure 2A**). When the mice were challenged with intradermal LPS there was an insignificant reduction in total bone marrow cells in both control and MN mice (**Figure 2A**). Challenge with intradermal *L. donovani* infection led to increased bone marrow cellularity in control mice, but this response was absent in MN mice. (**Figure 2A**). However, histopathological analysis of bone marrow cryosections revealed a striking difference in the cellular composition of the bone marrow in control and MN mice at baseline and following inflammatory challenge. At baseline, MN mice had significantly more lipid-containing (oil-red O positive) cells in the bone marrow hematopoietic centers compared to control mice (**Figure 2B**). Seventy-two hrs after challenge with or intradermal *L. donovani* (**Figure 2C**) or intraperitoneal LPS (**Figure 2D**) the difference in the number of fat cells in the bone marrow of MN and control mice was even more striking. Histopathological analysis confirmed that malnourished mice challenged with *L. donovani* or LPS had a significant expansion of the adipose area in the bone marrow compared with non-challenged malnourished controls (**Figure 2E**). When the oil-red O positive area in bone marrow was analyzed, LPS-induced lipid accumulation was amplified in MN mice. However, bone marrow adiposity was enhanced in the *L. donovani* challenge irrespectively of the nutritional status.

BM mesenchymal cells (MSC) can differentiate into either adipocytes or osteoblasts (54). Adipocytes in bone marrow can be identified by staining intracellular lipid with the lipophilic dye Oil-red O and osteoblasts and osteocytes are identified by staining for Alkaline Phosphatase (ALP). Although the presence of lipid droplets is often used as the defining characteristic of adipocytes, the storage of lipid droplets within cells of other lineages is well documented. In fact, lipid storage within osteoblasts or cells differentiating within the osteoblast lineage also occurs (54). Lipid accumulation may contribute to the energy needs of the cell but can also compromise bone mineral density if it exceeds the energy requirements of the cell (54). Because the cells identified in these studies as being oil-red-O positive have not been fully characterized we refer to them as lipid-filled or fat-containing cells rather than definitive adipocytes.

**Figure 3:**
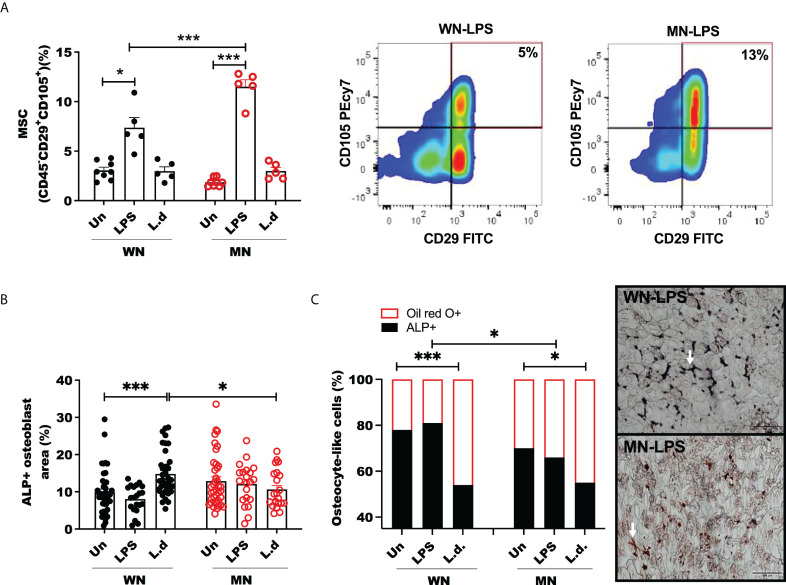
Mesenchymal stem cells are increased but osteoblasts unchanged in LPS challenged malnourished mice. Well-nourished (WN) or malnourished (MN) mice were challenged with LPS or *L. donovani* (*Ld*) and compared with naïve unchallenged (Un) mice. *L. donovani*-challenged mice were evaluated at 72h post-challenge and LPS-challenged mice were evaluated at 24h post-challenge. **(A)** Percentage of bone marrow mesenchymal cells (MSC) (CD45^-^CD29^+^CD115^+^) relative to total bone marrow cells and representative plot by flow cytometry (gated as **S Figure 1**). **(B)** Percentage of alkaline phosphatase positive area (ALP+) to identify osteoblasts in sternum cryosections. **(C)** Proportion of osteocyte-like cells in trabecular bone stained with Oil-Red O (Oil-red O +) or alkaline phosphatase (ALP+) in sternum cryosections. Right: Image showing osteocyte-like cells (white arrow) in trabecular bone double-stained with Oil-red O (red: lipid+) and alkaline-phosphatase (ALP+) (black: osteocyte), in sternum cryosections from LPS challenged mice. N=5 mice per group, 2 experiments.*p<0.05, ***p<0.001.

We investigated the balance of MSCs, lipid-filled cells, and osteoblasts in malnourished mice with and without inflammatory challenge. We found that the proportion of MSCs was not altered in naive (unchallenged) MN mice (**Figure 3A**), however, it increased significantly in control and malnourished mice 24h after challenge with LPS (**Figure 3A**; **Supplementary Figure 2B**). The LPS-induced increase in MSCs was amplified in MN mice, but the proportion of MSC was constant in *L. donovani*-challenged mice (**Figure 3A**). We then evaluated osteoblasts and osteocytes, which derive from MSCs, following exposure to the inflammatory stimulus. We found that the bone marrow area occupied by ALP+ cells (osteoblasts) was not altered by LPS challenge in either control or MN mice but *L.* donovani challenge increased the area occupied by ALP+ cells in control mice (**Figure 3B**). Expression of genes involved in osteogenesis, such as Dentin matrix acidic phosphoprotein 1 (Dmp1), Runt-related transcription factor 2 (Runx2), Osteocalcin (Bglap), and Osterix (Osx), showed a trend toward a decrease in LPS-challenged MN mice compared with naïve MN mice, but this difference was not observed in well-nourished mice (**Supplementary Figures 3A, B**). This support that a lack of osteoblast differentiation in the challenge infection is a consequence of malnutrition. The osteogenesis expression in malnourished naïve mice was variable but was particularly vulnerable to inhibition by the challenge (**Supplementary Figures 3A, B**).

To determine the effect of malnutrition and inflammatory challenge on the balance of cells differentiated from the MSC lineage we double-stained bone marrow sections with oil-red O (adipogenesis) and ALP (osteogenesis). We observed that osteoblasts/osteocytes (ALP+) in trabecular bone contained more lipid in malnourished LPS-challenged mice (**Figure 3C**). *L. donovani* infection enhanced the lipid content of osteocytes independently of the nutritional status (**Figure 3C**).

**Figure 4:**
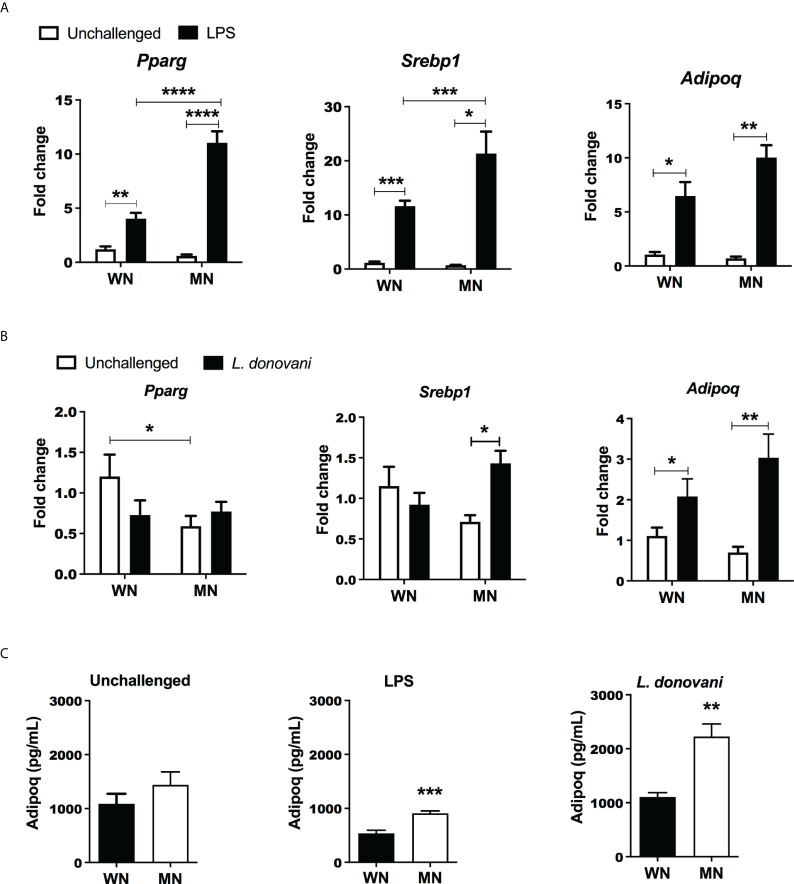
Malnutrition promotes expression of genes associated with adipogenesis following inflammatory challenge. **(A)** Expression of genes associated with adipocyte differentiation [Peroxisome Proliferator Activated Receptor gamma (*Pparg*), Sterol regulatory element binding transcription factor 1 (*Srebp1*), and Adiponectin (*Adipoq*)] in the bone marrow of naïve well-nourished (WN) and malnourished (MN) mice challenged with LPS and evaluated at 24h post-challenge. Data are shown as the mean fold change expression compared to naïve unchallenged (Un) mice. **(B)** Gene expression associated with adipocyte differentiation (*Pparg*, *Srebp1, Adipoq*) in the bone marrow of well-nourished (WN) and malnourished (MN) mice challenged with *L. donovani* and evaluated at 72h post-challenge. Determined by RT-qPCR. **(C)** Adiponectin protein levels in bone marrow lysates of well-nourished (WN) and malnourished (MN) mice (n=4 mice per group) unchallenged (UN) or challenged with LPS or *L. donovani* (*Ld*). LPS-challenged mice were evaluated at 24h post-challenge and *L. donovani*-challenged mice were evaluated at 72h post-challenge. Determined by Luminex and expressed as the mean concentration (pg/mL). N=5 mice per group, 2 experiments. **p<0.05, **p<0.01, ***p<0.001, ****p<0.0001.

Since the increase in MSCs was paralleled by increased cellular lipid content, we considered that MSC may be driven to differentiate toward adipocytes. In support of this, we found that the BM from mice challenged with LPS showed increased expression of genes involved in adipogenesis, peroxisome proliferator-activated receptor gamma (*Pparg*), sterol regulatory element-binding protein 1 (*Srebp1)*, Adiponectin (*Adipoq*) compared to unchallenged mice (**Figure 4A** **).** Following LPS challenge the increased expression of genes *Pparg* and *Srebp1* was amplified in MN mice compared to well-nourished (WN) mice (**Figure 4A**). *L. donovani* challenge followed a similar trend but a synergistic effect of malnutrition and *L. donovani* challenge was not significant **(** **Figure 4B**). We also found that the Bone marrow production of the adipokine, adiponectin, was also increased in both LPS- and *L. donovani*-challenged MN mice (**Figure 4C**).

### Malnutrition leads to increased generation and activation of osteoclasts

**Figure 5:**
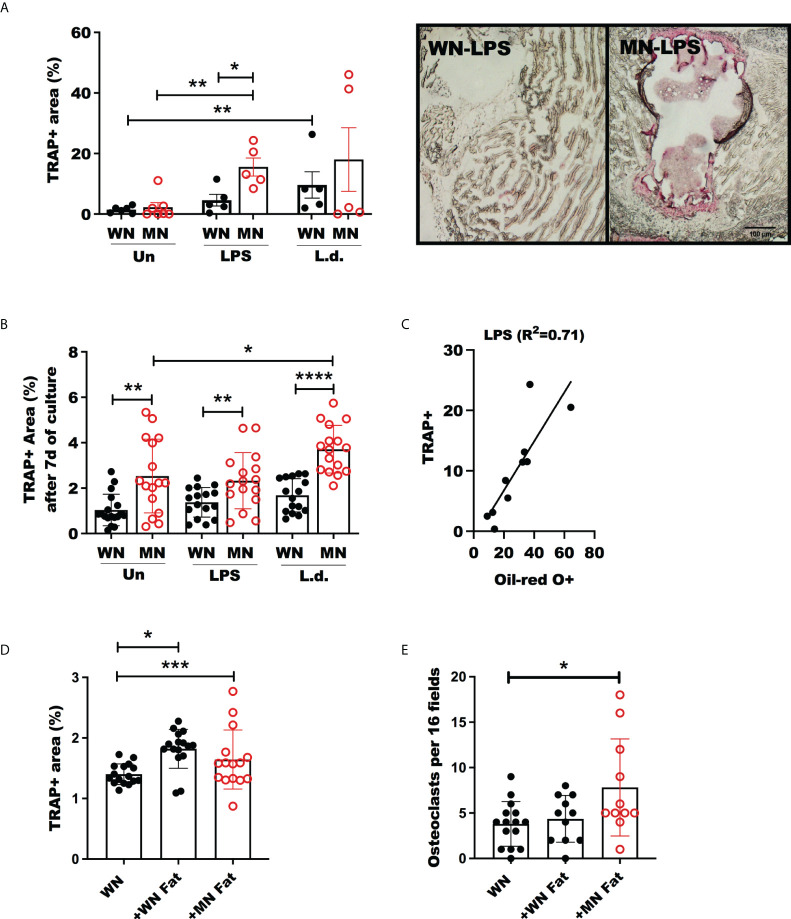
Increased Osteoclast activity in malnourished mice following inflammatory challenge. Well-nourished (WN) or malnourished mice (MN) remained naive unchallenged (Un) or were challenged with LPS or *L. donovani* (*Ld*). **(A)** Osteoclast activity measured by tartrate resistant acid phosphastase (TRAP) positive area in sternum cryosections and image showing TRAP staining (pink areas) of sternum cryosections from LPS challenged mice (same magnification for both panels). n=5 mice per groug, 2 experiments. **(B)** Percentage of area occupied by TRAP+ osteoclasts from femur bone marrow differentiated *ex vivo* with m-Csf and Rankl with or without exposure to LPS or *L. donovani* antigens (*L.d.*). **(C)** Linear correlation and correlation coefficient (R^2^) of TRAP+ area vs. oil-red-O+ area in sternum cryosections of LPS-challenged mice. **(D, E)** Percentage of area occupied by TRAP+ osteoclasts and number of osteoclasts per field from bone marrow femur of well-nourished mice (WN) differentiated with *ex vivo* with M-CSF and RANKL with or without exposure to 6mg/mL fat homogenate from well-nourished (+WN fat) or from malnourished mice (+MN fat). Number of multinucleated osteoclasts by Methyl green staining. Representative of 2 experimets with cells pooled from 2-3 mice/group. *p<0.05, **p<0.01, ***p<0.001, ****p<0.0001.

**Figure 6:**
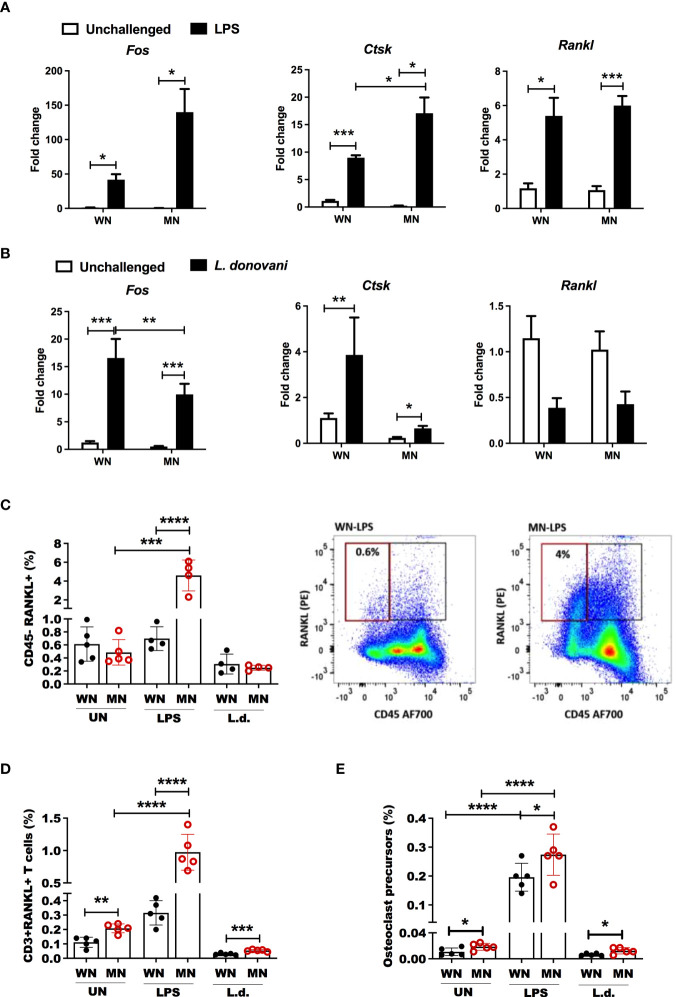
Malnutrition promotes expression of genes and cell populations associated with osteoclastogenesis following inflammatory challenge. **(A)** Expression of the osteoclast differentiation genes, Fos Proto-Oncogene (*Fos*), cathepsin (*Ctsk*) and RANK ligand (*Rankl*), in the bone marrow of well-nourished (WN) or malnourished (MN) mice that were naïve unchallenged (Un) or mice challenged with LPS. Bone marrow was obtained 24h after LPS challenge and expression determined by qRT-PCR. **(B)** Expression of *Fos*, *Ctsk*, and *Rankl* in the bone marrow of well-nourished (WN) or malnourished (MN) mice that were naïve unchallenged (Un) or challenged with *L. donovani* (*L.d*.). Bone marrow was obtained 72 hr after Ld challenge and expression determined by qRT-PCR. **(C)** Percentage of bone marrow non-hematopoietic cells expressing osteoclast differentiation/activation factor Rankl (CD45- Rankl+) relative to total cells in well-nourished (WN) or malnourished (MN) mice that were naïve unchallenged (Un) or challenged with LPS or *L. donovani* (*L.d*.). Bone marrow from LPS-challenged mice was obtained at 24h post-challenge and *L. donovani*-challenged mice at 72h post-challenge determined by flow cytometry. Right: representative dot-plot showing percentage of CD45-RANKL+ cells (gated as **S Figure 1B**). **(D)** Percentage of T cells expressing RANKL (CD3+RANKL+) in the bone marrow using flow cytometry (gated as **S Figure 1C**). **(E)** Percentage of bone marrow osteoclast precursors (CD45-CD115+ cKIT+) relative to total bone marrow cells in in well-nourished (WN) or malnourished (MN) mice that were naïve unchallenged (Un) or challenged with LPS or *L. donovani* (*L.d*.) determined by flow cytometry (gated as **S Figure 1D**). N=5 mice per group, 2 experiments. *p<0.05, **p<0.01, ***p<0.001, ****p<0.0001.

Osteoclasts are generated by differentiation from BM myeloid progenitors, which are derived from hematopoietic stem cells (55). Since osteoclasts antagonize the function of osteoblasts by participating in bone resorption, we evaluated the effect of malnutrition in osteoclast activation using Tartrate-Resistant Acid Phosphatase (TRAP) as the marker of osteoclasts. We found that LPS-induced TRAP activation of osteoclasts in malnourished mice **(** **Figure 5A** **)**. Likewise. the proportion and the total number of mature multinuclear osteoclasts were significantly increased (**Supplementary Figure 2A**). A similar trend of TRAP activation was observed in *L. donovani* challenged mice, but the difference was not significant. To confirm these results, we differentiated *ex vivo* bone marrow cells from control and MN mice to osteoclasts with Rankl and Csf-1 and exposed them to LPS or *L. donovani* antigen. We found increased osteoclast activation (TRAP+ cells) in the cells isolated from malnourished mice whether or not they had been challenged with LPS or *L donovani* (**Figure 5B**). Since the expansion of BM osteoclasts correlated strongly with the expansion of BM adiposity in LPS-challenged malnourished mice (**Figure 5C**), we exposed bone marrow cells from control mice to lysates from white fat adipose tissue collected from control or malnourished mice. Exposure to fat cell lysates increased the generation of TRAP+ osteoclasts irrespective of the nutritional status of the lipid donor (**Figure 5D**). However, exposure to malnourished fat resulted in increased numbers of mature multinuclear osteoclasts (**Figure 5E**). To further support the finding that osteoclastogenesis is promoted by malnutrition, we evaluated the expression of genes involved in osteoclastogenesis, Fos Proto-oncogene (*Fos)*, cathepsin (*Ctsk*) and Receptor Activator of NF-κB Ligand (*Rankl*) in malnourished mice with and without inflammatory challenge. We found that LPS-induced *Fos* and *Ctsk* expression was amplified in malnourished mice whereas *Rankl* expression was increased equally in WN and MN mice (**Figure 6A**). In contrast, *L. donovani* challenge induced *Fos* and *Ctsk*, but not *Rankl*, irrespective of the nutritional status (**Figure 6B**). We further analyzed the cell surface expression of RANKL by flow cytometry in the bone marrow since it is critical in the differentiation of bone-resorbing osteoclasts (31, 55). We found expansion of non-hematopoietic (CD45^_^) cells expressing RANKL in the bone marrow of naïve and challenged MN mice (**Figure 6C** **;** **SFigure 2C**). We also found that LPS-induced Rank+ T cell expansion (CD3+Rankl+ cells) in MN mice (**Figure 6D** **;** **SFigure 2D, E**). Additionally, we found that the percentage of osteoclast myeloid precursors was increased after LPS challenge in both WN and MN mice, but the increase was greater in malnourished mice (**Figure 6E** **;** **SFigure 2F**). Collectively, these data suggest that the bone marrow of MN mice exposed to an inflammatory challenge has increased differentiation of MSCs toward lipid-filled or adipocyte-like cells and that the adipogenic bone marrow environment is associated with osteoclast expansion/activation.

### Malnutrition leads to an exaggerated inflammatory response in the bone marrow

**Figure 7:**
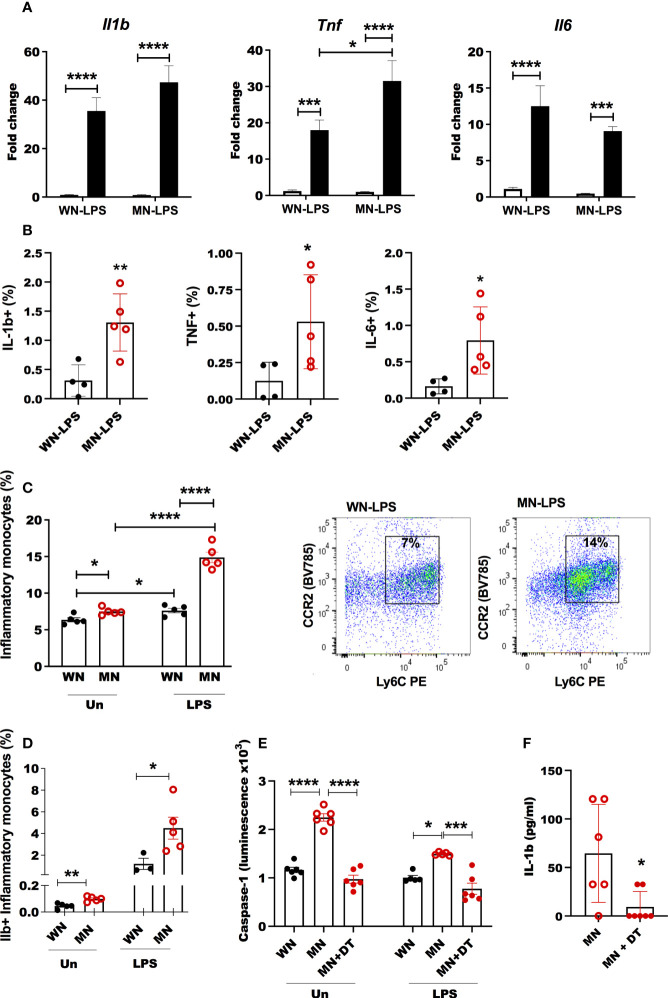
Heightened inflammatory response in bone marrow of malnourished mice following inflammatory challenge. **(A)** Expression of inflammatory cytokines interleukin-1β (*Il-1b*), tumor necrosis factor (*Tnf*) and interleukin-6 (*Il6*) in bone marrow of well-nourished (WN) or malnourished (MN) mice that were naïve unchallenged (Un) or challenged with LPS. Bone marrow was obtained 24h after LPS challenge and expression (fold change) relative to unchallenged WN mice, determined by qRT-PCR. **(B)** Percentage of IL-1ß, TNF and IL-6 in inflammatory monocytes in LPS challenged mice by flow cytometry. **(C)** Percentage of inflammatory monocytes (CD11b+Ly6C^int/hi^ Ly6G-CD11c-) relative to total bone marrow cells in well-nourished (WN) or malnourished (MN) mice that were naïve unchallenged (Un) or challenged with LPS or *L. donovani* (L.d.) determined by flow cytometry. Right: representative dot-plot showing inflammatory monocytes (CCR2+Ly6Chi/int) in LPS challenged mice, (gated as **Supplementary Figure 1E**). **(D)** Proportion of inflammatory monocytes containing Il-1ß naïve unchallenged (Un) or challenged mice with LPS by flow cytometry. **(E)** Inflammasome activation in bone marrow cells from Csfr1-LysMcre-DT malnourished (MN) mice treated or not with diphtheria toxin (+DT) to deplete inflammatory monocytes, compared with Csfr1-LysMcre-DT well-nourished mice (WN) after 1h of *in vitro* exposure to LPS or left unchallenged (Un); determined by luminometry with Caspase Glo-1 inflammasome assay. **(F)** Active Il-1ß in the bone marrow of Csfr1-LysMcre-DT malnourished (MN) mice challenged with LPS treated or not with diphtheria toxin (+DT) to deplete inflammatory monocytes. Determined by luminex. N=5 mice per group, 2 experiments. *p<0.05, **p<0.01, ***p<0.001, ****p<0.0001.

We evaluated the expression of bone marrow inflammatory cytokines (*Il6*, *Il1b*, and *Tnf*) that could have a role in shaping bone marrow adipogenesis and osteoclastogenesis in malnourished mice. The expression of *Il1b* and *Tnf* following LPS challenge was amplified in malnourished mice (**Figure 7A**), whereas malnutrition blunted LPS-induced *Il6* expression (**Figure 7A**). *L. donovani* challenge upregulated *Il1b* and *Tnf* but this effect was not affected by nutritional status (**Supplementary Figure 4**). Additionally, LPS-challenged MN mice had a significantly higher percentage of inflammatory monocytes in the bone marrow compared to LPS-challenged control mice (**Figure 7C** **;** **Supplementary Figure 2G**). This difference was not seen following *L. donovani* challenge. The percentage of cells that expressed inflammatory cytokines IL-1β, IL-6, and TNF significantly increased in the bone marrow of malnourished mice as determined by intracellular cytokine staining (**Figure 7B**). Similarly, the percentage of inflammatory monocytes that expressed IL-1β increased in LPS challenged and naïve mice (**Figure 7D**; **Supplementary Figure 5A**). Since IL-1β and inflammasome activation are known to promote bone resorption under inflammatory conditions (56, 57), we evaluated inflammasome activation in inflammatory monocytes in the bone marrow of malnourished mice using caspase-1 activation as a readout. We used *Csfr1*-DT transgenic mice in which the diphtheria toxin (DT) receptor is selectively expressed in monocytes under control of the *Csfr1* promoter (49). Inflammasome activation was significantly increased in malnourished naïve mice compared with control naïve mice. This difference was maintained but not amplified by *ex vivo* exposure of the bone marrow cells to LPS (**Figure 7E**). Depletion of inflammatory monocytes by administration of DT to malnourished mice (**Supplementary Figure 5B**) reduced bone marrow expression of caspase-1, and Il-1β in LPS challenged mice confirming their role in malnutrition-related inflammasome activation (**Figures 7E, F**). However, despite IL-1β and adiponectin being reduced concomitantly with inflammatory monocytes in DT mice (**Supplementary Figure 5C**), TNF was increased and IL-6 was not modified (**Supplementary Figure 5C**). Suggesting inflammation-driven independently of inflammatory monocytes.

### Dietary fish oil reduces inflammation and osteoclastogenesis in malnourished mice

**Figure 8:**
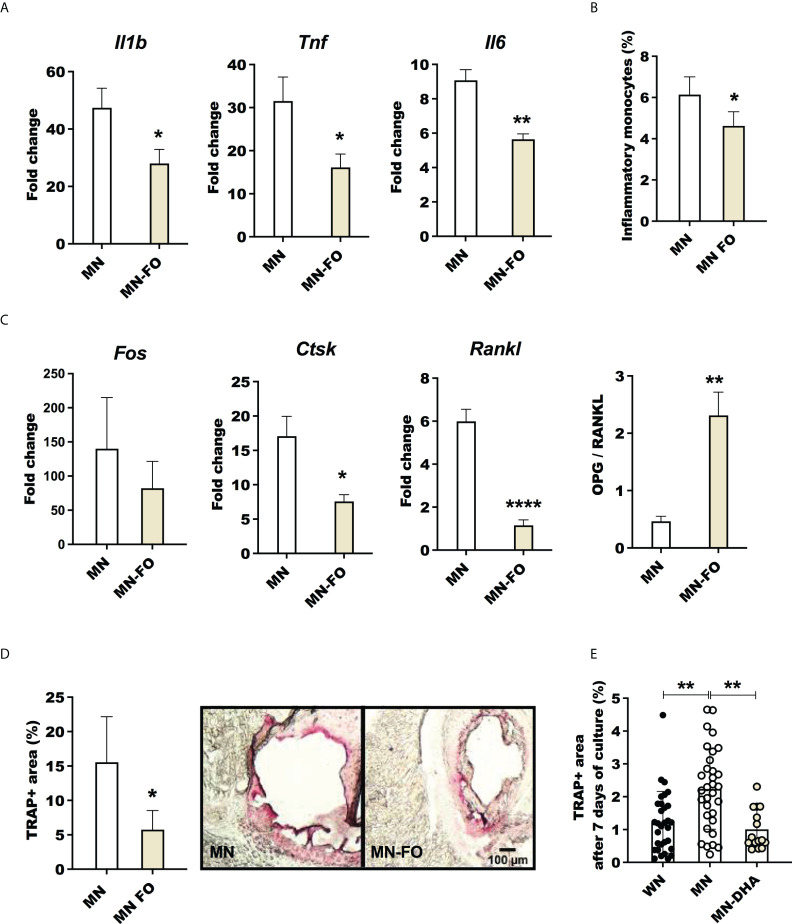
Fish oil supplementation decreases inflammation and osteoclast activation in malnourished mice following inflammatory challenge. Malnourished were mice fed with the standard nutrient deficient diet containing corn oil (MN) or with a nutrient deficient diet in which the corn oil was replaced by fish oil (MN-FO). Both groups of mice were challenged with LPS and the bone marrow isolated 24 hrs later. **(A)** Expression of interleukin-1β (*Il1b*), tumor necrosis factor (*Tnf*) and interleukin-6 (*Il6*) in bone marrow. Determined by qRT-PCR and expressed as the fold-change over unchallenged mice from the same group. **(B)** Percentage of inflammatory monocytes (CD11b+Ly6C^int/hi^ Ly6G-CD11c-) relative to total bone marrow cells determined by flow cytometry (gated as shown in **Supplementary Figure 1E**). **(C)** Expression of genes associated with osteoclastogenesis, *Fos*, *Ctsk* and *Rankl*, and ratio of the expression of *Opg*/*Rankl* in the bone marrow. **(D)** Percentage of area occupied by tartrate-resistant acid phosphatase positive (TRAP+) in sternum cryosections image of a TRAP+ field in the bone marrow (pink area). **(E)** Percentage of area occupied by TRAP+ osteoclasts after 7 days of differentiation of bone marrow cells from LPS challenged mice, treated *ex vivo* with the omega-3 fatty acid Docosahexaenoic acid (MN-DHA) or vehicle (MN) and compared with those from well-nourished mice (WN). N=5 mice per group, 2 experiments. *p<0.05, **p<0.01.

Lipid-based nutrient supplements are used in the treatment of malnourished children because of their high energy density (58). These supplements are typically high in omega-6 fatty acids (59), which are pro-inflammatory (60). Similarly, the nutrient-deficient mouse chow used in our malnutrition model has corn oil as the lipid source, which is rich in pro-inflammatory omega-6 fatty acids. In contrast, dietary omega-3 fatty acids have anti-inflammatory effects (61). Since dietary fish oil (rich in omega-3) supplementation has been associated with an increased omega-3 to omega-6 ratio (62) and decreased inflammation (60, 61), we reasoned that incorporation of omega-3-rich fish oil in a nutrient-deficient diet would decrease inflammation and osteoclast activation compared with the standard omega-6-rich nutrient-deficient diet. Indeed, mice fed the nutrient-deficient diet with fish oil rather than corn oil as the lipid source showed reduced expression of inflammatory cytokines (*Il1b*, *Tnf*, and *Il6*) (**Figure 8A**) and a decreased percentage of inflammatory monocytes (**Figure 8B**) in the bone marrow following LPS challenge. LPS-challenged malnourished mice that received dietary fish oil also showed downregulation of genes associated with osteoclastogenesis (*Ctsk* and *Rankl*) (**Figure 8C**). The ratio of expression of osteoprotegerin (*Opg*) to *Rankl*, which is indicative of bone integrity, was significantly increased in mice that received the nutrient-deficient diet with fish oil (**Figure 8C**). We also found fewer TRAP+ activated osteoclasts in the bone marrow of malnourished mice that received the fish oil (**Figure 8D**), which was indicative of decreased bone resorption in fish-oil-fed mice. Lastly, to confirm an effect of omega-3 fatty acids in suppressing osteoclastogenesis we found fewer TRAP+ osteoclasts in bone marrow cultures from LPS- and *L. donovani*-challenged malnourished mice exposed *ex vivo* to Docosahexaenoic acid (DHA) (**Figure 8E**). Similarly, bone marrow from *L. donovani*-challenged malnourished mice showed reduced differentiation to TRAP+ osteoclasts when cultured *ex vivo* in the presence of DHA (**Supplementary Figure 6D**). Dietary fish-oil mediated reduction in expression of proinflammatory cytokines and genes associated with osteoclastogenesis paralleled the reduction in osteoclast activity in LPS-challenged malnourished mice, this was not observed in *L. donovani*-challenged mice (**Supplementary Figures 6A, B**). Collectively, these data indicate that malnutrition-related inflammation and osteoclastogenesis can be blunted by dietary omega-3 fatty acids.

## Discussion

This study investigated the interaction between malnutrition and inflammatory challenge on bone marrow composition and bone health. Malnutrition and inflammatory challenge synergistically increased bone marrow MSCs and skewed differentiation of MSCs toward lipid-filled cells/adipocytes rather than bone-forming osteoblasts. This compromise in bone-building capacity was accompanied by increased bone-resorbing osteoclast activity, which together would contribute to impaired bone growth and strength. These changes in BM composition were associated with an enhanced bone marrow inflammatory response and caspase-1-driven IL-1ß activation in inflammatory monocytes. Replacing dietary pro-inflammatory omega-6-rich lipids (corn oil) with anti-inflammatory omega-3-rich lipids (fish oil) (60, 63, 64), blunted the bone marrow inflammatory response and osteoclastogenesis.

The rapid expansion of bone marrow adiposity in malnourished mice exposed to an acute inflammatory (LPS) or infectious (*L. donovani*) challenge was associated with an increase in bone marrow MSCs (precursors of adipocytes) and a transcriptional signature consistent with adipocyte differentiation. Bone marrow adipocyte differentiation from MSCs and bone homeostasis is regulated by the transcription factor PPARγ (65, 66) whose expression we found to be synergistically increased in the bone marrow of LPS-challenged MN mice. This transcriptional program that favors adipocyte over osteoblast differentiation, would lead to increased bone marrow adiposity at the expense of bone formation. The increased bone marrow expression of PPARγ could be a consequence of the NFKB activated through pro-inflammatory cytokines in concert with the mediator PGE_2_ (67), which is elevated in childhood malnutrition (68) and in our mouse model (37–39).

The role of bone marrow adiposity in health and disease is still not clear, but increased adiposity in the malnourished host could have several consequences. First, differentiation of bone marrow MSCs toward adipocytes and away from bone-forming osteoblasts would weaken bone strength and reduce bone length. Although our model is most relevant to acute malnutrition, similar changes in bone marrow composition could also contribute to the linear growth faltering of childhood stunting. Second, expansion of adiposity is likely to lead to accumulation of lipids that are subject to oxidation and glycation, which enhance inflammatory responses (69) and would further promote *Pparg* and *Rankl* expression, osteoclast activity, and bone loss (66, 70). Our *in vitro* experiments showed that exposure of bone marrow cells to products from adipose tissue from malnourished mice enhanced the *ex vivo* differentiation of mature osteoclasts. Adipose tissue and its metabolites such as lipotoxic fatty acids ceramides, cholesterol, and oxidized low-density lipoprotein have been shown to activate the Nlrp3, inflammasome, and caspase-1, driving osteoclast differentiation and activation (71, 72). In some cases, this activation is independent of inflammation. Ceramides of *Porphyromonas gingivalis* promote RANKL-induced osteoclastogenesis independently of TLRs or inflammasome activation (73), supporting the concept that alteration of the lipid balance associated with inflammation is enough to drive enhanced osteoclastogenesis. Third, while bone marrow adipocytes produce hormones that support hematopoietic development (74), under some circumstances excessive expansion of bone marrow adiposity may negatively regulate hematopoiesis (75). Lastly, expansion of bone marrow adipocytes could also benefit the host. Replacement of metabolically active hematopoietic tissue with fatty tissue could be a mechanism used by the host to minimize energy requirements during the malnourished state (76). Other studies have shown the importance of lipids in bone homeostasis, as well as the requirement of fatty acid oxidation for the normal function and development of osteoblasts (77).

The heightened inflammatory response in the bone marrow of malnourished mice was associated with a larger TRAP+ osteoclast cell mass and increased differentiation and activation of osteoclasts in *ex vivo* cultures. Other studies similarly showed that LPS and other microbial products induced the differentiation of osteoclasts (78). Inflammatory cytokines drive osteoclast activation with subsequent bone loss characteristic of osteolytic diseases such as periodontitis and rheumatoid arthritis (79). It is well established that inflammatory cytokines, such as IL-1β, TNF, IL-17a, and IL-6 cooperate with RANKL to induce osteoclast differentiation (73, 78, 80). The synergistic effect of malnutrition and inflammatory challenge on *Rankl* expression found in our study is consistent with those findings. While other pathogens may promote the development of osteoclasts and bone osteolytic activity (81, 82), we found *L. donovani* was a weak inducer of osteoclastogenesis relative to LPS. The less-intense osteoclast activation after *Leishmania* challenge could be explained by the distant site of inoculation with few parasites reaching the bone marrow and/or parasite stealth and suppressive mechanisms that dampen the early TNF, IL-1β, and IL-6 inflammatory response (83, 84). Nevertheless, our *in vitro* studies showed that *Leishmania* antigens were able to induce osteoclast activation. Despite the limited effect of acute *L. donovani* challenge on the bone marrow and bone in this model, it is likely that chronic visceral leishmaniasis, which is characterized by a proinflammatory cytokine storm (85), would likely lead to bone resorption. This notion is supported by evidence of bone tissue destruction in Balb/c mice chronically infected with *L. amazonensis* (86).

We found evidence of inflammasome activation in the BM of malnourished mice with a high proportion of inflammatory monocytes expressing IL-1β BM caspase-1 activation. Other studies demonstrated that RANK and IL1-β regulate osteoclastogenesis and bone resorption *via* calcineurin (57, 87). Bacterial products also activate the inflammasome components NLRP3 and AIM2 to release mature IL-1β (88). The activation of inflammasome components Nlrp3, Nlrc4, and IL-1β results in bone necrosis and bone resorption (56). Inflammasomes can be activated in a low-grade inflammation typical of non-infectious insults such as aging, promoting osteoclast differentiation, bone loss, and osteolysis (89). Our finding of high inflammasome activation in the bone marrow of naïve malnourished mice supports the notion that malnutrition promotes a state of sustained inflammation (10, 90) that favors bone resorption. Despite the inflammasome activation and IL-1ß production being dependent on inflammatory monocytes in DT malnourished mice, TNF and IL-6 were not abrogated suggesting that inflammatory monocytes are not the sole source of inflammatory cytokines in this model.

Long-chain omega-3 polyunsaturated fatty acids have anti-inflammatory properties (60). A high omega-6 to omega-3 ratio is associated with chronic inflammatory diseases (91). In experimental periodontitis, omega-3 metabolites decreased bone resorption through the inhibition of inflammatory cell infiltration and cytokine production (92). Our study also showed that dietary omega-3-rich fish oil decreased the expression of inflammatory cytokines (*Il1b, Tnf, Il6*) in LPS-challenged malnourished mice. Since the pro-inflammatory PGE_2_, a product of omega-6 fatty acids, is elevated in human and mouse malnutrition (39, 68) it may have a role in the heightened inflammation of malnourished mice found in this study. In agreement with this, aged mice fed with fish oil showed a decreased level of pro-inflammatory cytokines and improved bone density through decreased *Cox-2* and *Pparg* activation (64). In addition to inflammation, omega-3 could be acting through the inhibition of the differentiation of osteoclasts. We found that LPS-challenged malnourished mice that received dietary omega-3 down-regulated the expression of *Rankl*, *Fos*, and *Cstk*, which are crucial for osteoclast development through RANK signaling (87). Others showed that Omega-3 reduced the differentiation of osteoclasts in experimental periodontitis (93). Dietary omega-3 fatty acids may also decrease adipogenic capacity (64) and enhance the differentiation of osteoblasts, mediated by enhancement of the transcription factor *Runx2* and *Pparg* suppression (94). Inflammation-independent effects were also observed in *L. donovani* challenged malnourished mice where dietary fish oil reduced osteoclast activation without down-modulation of inflammatory cytokines. Together these findings suggest that modulation of the lipid balance with omega-3 fatty acids favors bone marrow function that supports bone health in the malnourished host.

In summary, we present experimental evidence demonstrating synergism between malnutrition and inflammation in mediating changes in bone marrow composition and bone health. Our results show that after inflammatory challenge, the bone marrow of malnourished mice compared to controls had increased inflammatory cytokine expression and caspase 1-dependent IL-1ß production in inflammatory monocytes, which could enhance osteoclast differentiation and activation. These together with a greater expansion of MSCs associated with the production of lipid-filled cells or adipocytes instead of bone-forming osteoblasts. Collectively these processes would decrease bone formation and promote bone resorption, contributing to growth faltering and bone fragility in malnourished children. Dietary omega-3-rich fish oil ameliorated malnutrition-related bone marrow inflammation and osteoclast differentiation and activation. These data support a previous recommendation (95) to revisit the formulation of lipid-based (omega-6 rich) nutritional supplements with consideration of the inclusion of omega-3 fatty acids for the treatment and prevention of childhood malnutrition.

## Data availability statement

The original contributions presented in the study are included in the article/**Supplementary Material**. Further inquiries can be directed to the corresponding authors.

## Ethics statement

The animals used in this study were handled in strict accordance with the recommendations in the Guide for the Care and Use of Laboratory Animals of the National Institutes of Health. The protocol was approved by the Institutional Animal Care and Use Committee of the University of Texas Medical Branch, Galveston, Texas (protocol number 1306027).

## Author contributions

EO, PM, and BT conceived the project and designed the experiments. EO, GP, GTP, EC, and AU-P performed the experiments. EO, EC, and GP analyzed the data. EO wrote the initial manuscript with all authors providing critical feedback. BT and ZG contributed fruitful discussions and helpful ideas. EO, PM, ZG, and BT made a critical revision of the manuscript. All authors contributed to the article and approved the submitted version.

## Funding

This work was supported by the U.S. National Institutes of Health (NIH/NIAID) grant number AI130126 to PCM. GEP was supported by NIH/NIAID training grant number 2T32AI007526. GP was supported by a fellowship from the International Mentoring Foundation for the Advancement of Higher Education (IMFAHE).

## Acknowledgments

The authors acknowledge the helpful contributions of Jessica Flowers, PhD, Lab Animal Nutritionist at Envigo for help with formulating the mouse diets, and the staff and veterinarians of the animal research center for help in the care of the mice.

## Conflict of interest

The authors declare that the research was conducted in the absence of any commercial or financial relationships that could be construed as a potential conflict of interest.

## Publisher’s note

All claims expressed in this article are solely those of the authors and do not necessarily represent those of their affiliated organizations, or those of the publisher, the editors and the reviewers. Any product that may be evaluated in this article, or claim that may be made by its manufacturer, is not guaranteed or endorsed by the publisher.

## Supplementary material

Supplementary Figure 1 Gating strategy. **(A)** Mesenchymal cells (MSC): cell population is gated in singlets (single events). Singlets are sub-gated in the live cell population (negative for UV); live cells are sub-gated to define CD45 cells labeled with Alexa fluor 700 (CD45 AF 700); CD45 negative cells (CD45- AF700) are sub-gated to a quadrant with the population CD105 (endoglin) positive (CD105+ PE Cy7) and CD29 positive (CD29+ FITC) (MSC: CD45-CD105+CD29+). **(B)** CD45-RANKL+ cells: singlets and live cells are sub-gated to define a quadrant with CD45 Alexa fluor 700 negative cells (CD45- AF 700) and RANKL PE positive cells (RANKL+ PE) (CD45-RANKL+). **(C)** CD3+ RANKL+ cells: singlets and live cells are sub-gated to a quadrant with CD45 cells Alexa fluor 700 positive (CD45+ AF 700) and CD3 cells Pacific Blue positive (CD3+ Pacific Blue). Double positive cells (CD45+CD3+ cells) are sub-gated to define RANKL positive cells (RANKL+ PE) in the CD3 positive population (CD45+CD3+RANKL+). **(D)** Osteoclast precursors: singlets and live cells are sub-gated to define CD45 positive cells (CD45+ AF700) CD45+ cells are sub-gated to define cells expressing cKIT (cKIT+ APCcy7). cKIT positive cells are sub-gated to a quadrant with CD115 positive cells (CD115+ SB 600) and cells CD11b low or negative (CD11b^low-^ PEcy7) (osteoclast precursor: CD45+cKIT+CD115+CD11b^low/-^). **(E)** Inflammatory monocytes: singlets and live cells are sub-gated to a quadrant with CD45 positive cells (CD45+ AF700) and the neutrophil marker Ly6G, to exclude neutrophils (LyG+ APC). The population CD45 positive Ly6G negative (CD45+Ly6G-) is sub-gated to a quadrant with CD11b (APC) and CD11c (eFluor506) to exclude CD11c+cells. The CD11b+CD11c- population is sub-gated to a quadrant with CCR2 BV785 and Ly6C (PE) positive cells. Inflammatory monocytes are CCR2+Ly6C^hi/int+^ (CD45+Ly6G-CD11c-CD11b+CCR2+Ly6C^hi/int+^).

Supplementary Figure 2 Percentage of multinucleated osteoclasts and number of cells in bone marrow. **(A)** Proportion of multinuclear osteoclast in the bone marrow of LPS challenged mice by nuclear staining with 7AAD and representative flow cytometry dot-plot. **(B)** Number of Mesenchymal cells (MSC: CD45-CD29+CD105+). **(C)** Number of CD45-Rankl+ cells. **(D)** Representative dot-plot showing proportion of T cells expressing Rankl (CD3+Rankl+) (gated as in **Supplementary Figure 1C**). **(E)** Number of T cells Rankl+ (CD45+CD3+ Rankl+). **(F)** Number of osteoclast precursors (CD45+cKIT+ CD115+Cd11b^low/-^). **(G)** Number of inflammatory monocytes (CD45+Ly6G-CD11c-CD11b+CCR2+Ly6C^hi/int+)^. Number of cells= proportion of cell population by flow cytometry x total number of cells counted by microscopy/100. Note that the increased number of cells in LPS-MN group is still noticeable despite Malnourished mice are smaller and tend to have lower total of cells recovered from the smaller femurs. Un, uninfected; L.d., infected with *L. donovani*. N=5 mice per group, 2 experiments.

Supplementary Figure 3 Expression of osteogenic genes in bone marrow of LPS and *L. donovani*-challenged mice. Dentin matrix acidic phosphoprotein 1 (Dmp1), Runt-related transcription factor 2 (Runx2), Osteocalcin (Bglap), and Osterix (Osx), in **(A)** Bone marrow of LPS challenged mice (24h); **(B)** *L. donovani*-infected mice (72h). Data are expressed as fold-change relative to naïve well-nourished unchallenged mice. N=5 mice per group.

Supplementary Figure 4 Expression of cytokines in bone marrow of *L. donovani*-challenged mice. Expression of *Il1b*, *Tnf* and *Il6* in bone marrow of well-nourished (WN) or malnourished (MN) mice that were naïve unchallenged (Un) or challenged with *L. donovani* (*L.d*.). Bone marrow was obtained 72h after LPS challenge and expression (fold change) determined by qRT-PCR relative to unchallenged WN mice. N=5 mice per group, 2 experiments.

Supplementary Figure 5 Contribution of inflammatory monocytes to bone marrow inflammation in malnourished mice. **(A)** Percentage of inflammatory monocytes positive for intracellular interleukin-1β (IL-1β), tumor necrosis factor alpha (TNF) and interleukin-6 (IL-6) in inflammatory monocytes (Ly6C^hi/int^, CD11b+, CD11c-Ly6G-) from naïve unchallenged well-nourished (WN) or malnourished mice (MN) by flow cytometry. *p<0.05, **p<0.01. **(B)** Proportion of inflammatory monocytes in bone marrow from CSFR1-LysMcre-DT malnourished (MN) mice untreated (No DT) or treated with diphtheria toxin (+DT) to deplete inflammatory monocytes and representative dot-plot. **(C)** Cytokines in the bone marrow of DT mice, determined by Luminex. N=5 mice per group, 2 experiments. (*p<0.05).

Supplementary Figure 6 Effect of fish oil supplementation on inflammation and osteoclast activation in malnourished mice following *L. donovani* challenge. Malnourished were mice fed with the standard nutrient deficient diet containing corn oil or with an identical nutrient deficient food in which the corn oil was replaced by fish oil (MN-FO). Both groups of mice were challenged with *L. donovani* and the bone marrow isolated for evaluation 72 hrs later. **(A)** Bone marrow expression of interleukin-1(*Il1b*), tumor necrosis factor alpha (*Tnf*) and interleukin-6 (*Il6*) Data are expressed as fold-change relative to naïve well-nourished unchallenged mice. **(B)** Bone marrow expression of genes associated with osteoclast activation in the bone marrow, Fos Proto-Oncogene (*Fos*), cathepsin (*Ctsk*) and RANK ligand (*Rankl*). Data are expressed as fold-change relative to naïve well-nourished unchallenged mice. **(C)** Percentage of area occupied by tartrate-resistant acid phosphatase positive (TRAP+) cells in in sternum cryosections. **(D)** Percentage of area occupied by TRAP+ osteoclasts after 7 days of *ex vivo* differentiation of bone marrow cells from *L. donovani-* infected mice treated *with* Docosahexaenoic acid (MN-DHA) or with vehicle (MN) and were compared with those from well-nourished mice (WN). N=5 mice per group, 2 experiments. *p<0.05, **p<0.01.
