# Supplementary figures and images for: Inflammatory stimuli alter bone marrow composition and compromise bone health in the malnourished host
Source: Front Immunol. 2022 Aug 2;13:846246. doi: 10.3389/fimmu.2022.846246 (PMC9380851; doi:10.3389/fimmu.2022.846246)

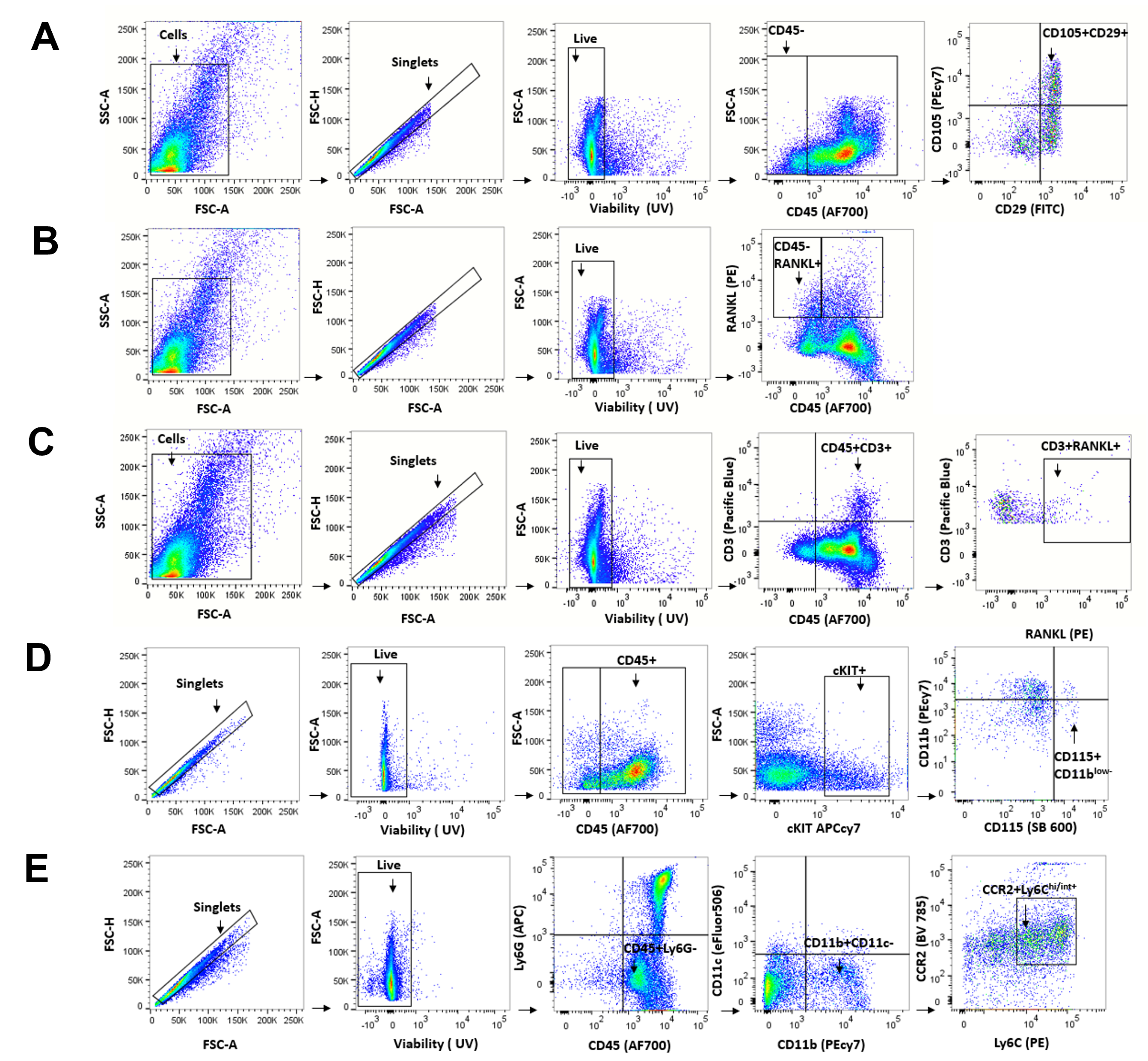

Supplement: Supplementary Figure 1 — Gating strategy. (A) Mesenchymal cells (MSC): cell population is gated in singlets (single events). Singlets are sub-gated in the live cell population (negative for UV); live cells are sub-gated to define CD45 cells labeled with Alexa fluor 700 (CD45 AF 700); CD45 negative cells (CD45- AF700) are sub-gated to a quadrant with the population CD105 (endoglin) positive (CD105+ PE Cy7) and CD29 positive (CD29+ FITC) (MSC: CD45-CD105+CD29+). (B) CD45-RANKL+ cells: singlets and live cells are sub-gated to define a quadrant with CD45 Alexa fluor 700 negative cells (CD45- AF 700) and RANKL PE positive cells (RANKL+ PE) (CD45-RANKL+). (C) CD3+ RANKL+ cells: singlets and live cells are sub-gated to a quadrant with CD45 cells Alexa fluor 700 positive (CD45+ AF 700) and CD3 cells Pacific Blue positive (CD3+ Pacific Blue). Double positive cells (CD45+CD3+ cells) are sub-gated to define RANKL positive cells (RANKL+ PE) in the CD3 positive population (CD45+CD3+RANKL+). (D) Osteoclast precursors: singlets and live cells are sub-gated to define CD45 positive cells (CD45+ AF700) CD45+ cells are sub-gated to define cells expressing cKIT (cKIT+ APCcy7). cKIT positive cells are sub-gated to a quadrant with CD115 positive cells (CD115+ SB 600) and cells CD11b low or negative (CD11blow- PEcy7) (osteoclast precursor: CD45+cKIT+CD115+CD11blow/-). (E) Inflammatory monocytes: singlets and live cells are sub-gated to a quadrant with CD45 positive cells (CD45+ AF700) and the neutrophil marker Ly6G, to exclude neutrophils (LyG+ APC). The population CD45 positive Ly6G negative (CD45+Ly6G-) is sub-gated to a quadrant with CD11b (APC) and CD11c (eFluor506) to exclude CD11c+cells. The CD11b+CD11c- population is sub-gated to a quadrant with CCR2 BV785 and Ly6C (PE) positive cells. Inflammatory monocytes are CCR2+Ly6Chi/int+ (CD45+Ly6G-CD11c-CD11b+CCR2+Ly6Chi/int+). [file Image_1.tif]

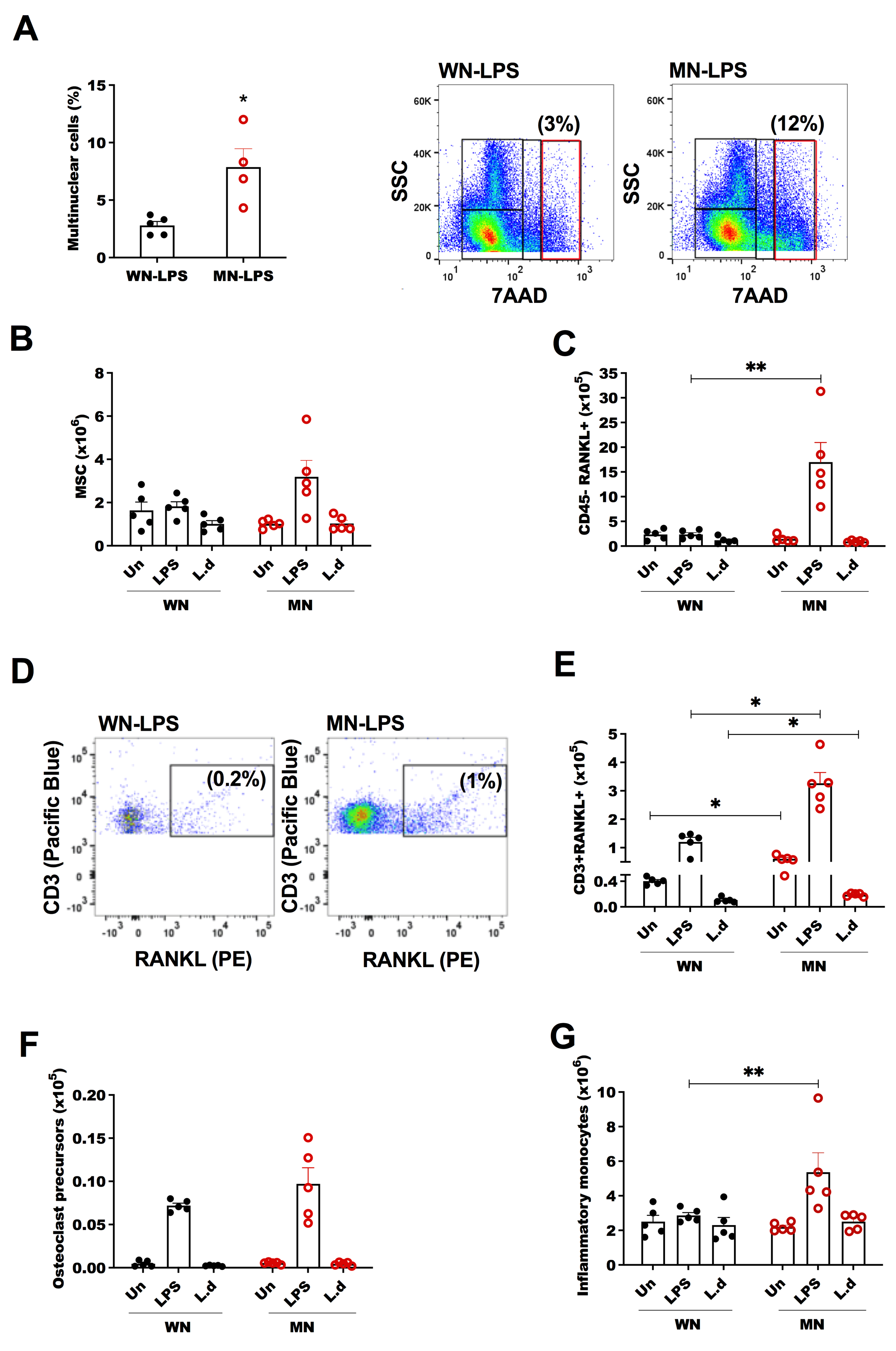

Supplement: Supplementary Figure 2 — Percentage of multinucleated osteoclasts and number of cells in bone marrow. (A) Proportion of multinuclear osteoclast in the bone marrow of LPS challenged mice by nuclear staining with 7AAD and representative flow cytometry dot-plot. (B) Number of Mesenchymal cells (MSC: CD45-CD29+CD105+). (C) Number of CD45-Rankl+ cells. (D) Representative dot-plot showing proportion of T cells expressing Rankl (CD3+Rankl+) (gated as in Supplementary Figure 1C). (E) Number of T cells Rankl+ (CD45+CD3+ Rankl+). (F) Number of osteoclast precursors (CD45+cKIT+ CD115+Cd11blow/-). (G) Number of inflammatory monocytes (CD45+Ly6G-CD11c-CD11b+CCR2+Ly6Chi/int+). Number of cells= proportion of cell population by flow cytometry x total number of cells counted by microscopy/100. Note that the increased number of cells in LPS-MN group is still noticeable despite Malnourished mice are smaller and tend to have lower total of cells recovered from the smaller femurs. Un, uninfected; L.d., infected with L. donovani. N=5 mice per group, 2 experiments. [file Image_2.tiff]

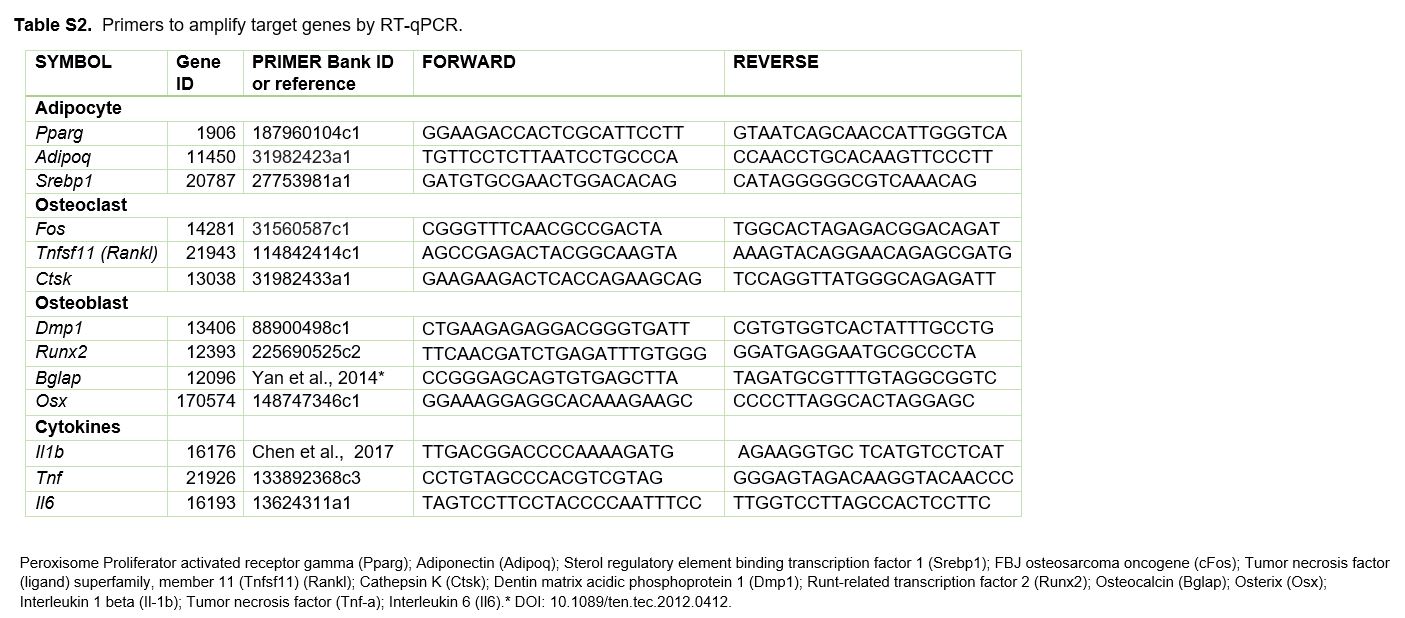

Supplement: Supplementary Figure 3 — Expression of osteogenic genes in bone marrow of LPS and L. donovani-challenged mice. Dentin matrix acidic phosphoprotein 1 (Dmp1), Runt-related transcription factor 2 (Runx2), Osteocalcin (Bglap), and Osterix (Osx), in (A) Bone marrow of LPS challenged mice (24h); (B) L. donovani-infected mice (72h). Data are expressed as fold-change relative to naïve well-nourished unchallenged mice. N=5 mice per group. [file Image_3.jpeg]

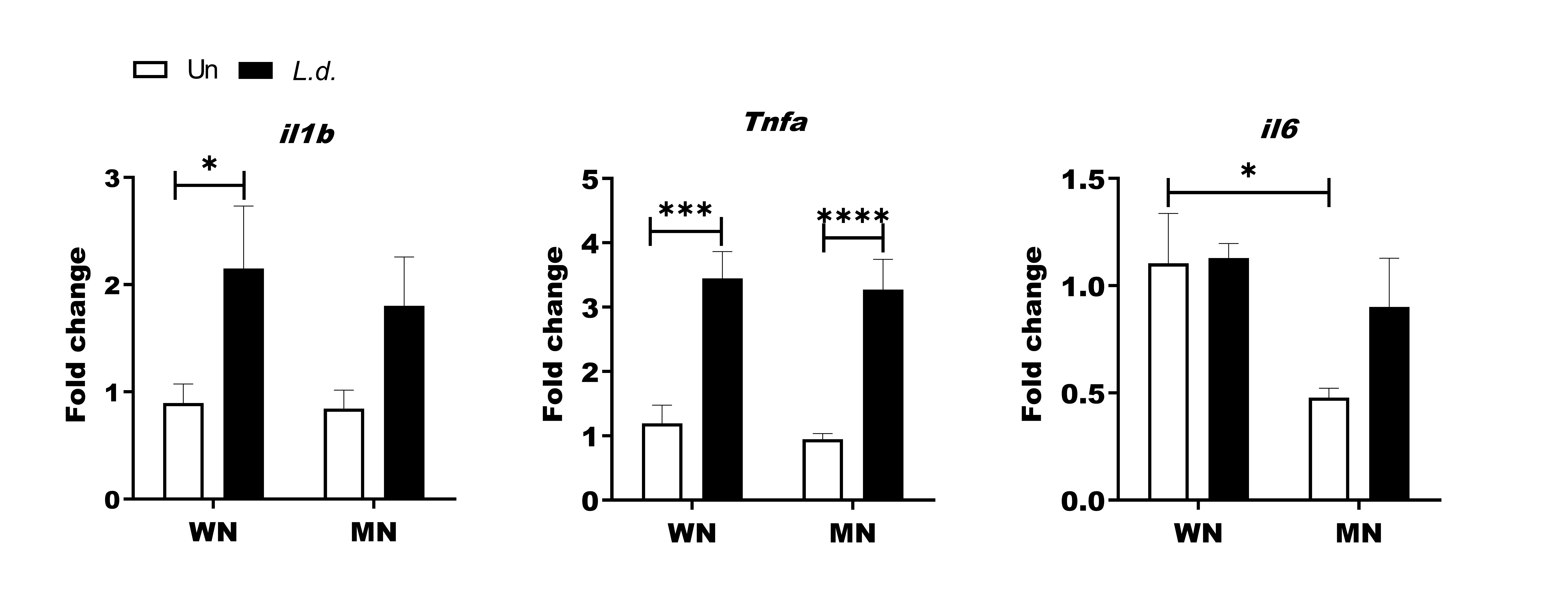

Supplement: Supplementary Figure 5 — Contribution of inflammatory monocytes to bone marrow inflammation in malnourished mice. (A) Percentage of inflammatory monocytes positive for intracellular interleukin-1β (IL-1β), tumor necrosis factor alpha (TNF) and interleukin-6 (IL-6) in inflammatory monocytes (Ly6Chi/int, CD11b+, CD11c-Ly6G-) from naïve unchallenged well-nourished (WN) or malnourished mice (MN) by flow cytometry. *p<0.05, **p<0.01. (B) Proportion of inflammatory monocytes in bone marrow from CSFR1-LysMcre-DT malnourished (MN) mice untreated (No DT) or treated with diphtheria toxin (+DT) to deplete inflammatory monocytes and representative dot-plot. (C) Cytokines in the bone marrow of DT mice, determined by Luminex. N=5 mice per group, 2 experiments. (*p<0.05). [file Image_5.tif]
