# Supplementary material for: Inflammatory stimuli alter bone marrow composition and compromise bone health in the malnourished host
Source: Front Immunol. 2022 Aug 2;13:846246. doi: 10.3389/fimmu.2022.846246 (PMC9380851; doi:10.3389/fimmu.2022.846246)
Supplement: Supplementary file 8 [file DataSheet_1.pdf]

Table S1. Anti-mouse antibodies to identify cell populations in the bone marrow by flow cytometry.

**target**	**clone**	**Brand**
**Monocytes**		
CD192 (CCR2)	SA203G11	Biolegend
CD11b	M1/70	Biolegend
Ly6C	HK1.4	Biolegend
**T cells inducing osteoclasts**		
CD3+	145-2C11,	Biolegend
CD254+ (RANKL+)	IK22/5	eBioscience
**Osteoclasts precursor**		
CD11b+^{low}	M1/70	Biolegend
CD45+	30-F11	BD Biosciences
CD117+ (c-kit+)	ACK-2	Biolegend
CD115+ (CSFR-1R+)	AFS98	eBioscience
**Mesenchymal cells**		
CD45-	30-F11	BD Biosciences
CD29+	Ha2/5	BD Biosciences
CD105+ (Endoglin)	MJ7/18	eBioscience
**Vital makers**		
LIVE/DEAD Fixable Green stain		ThermoFisher Sci.
Zombie UV<br>Ghost Dye		Biolegend<br>Tonbo biosciences
**Cytokines**		
TNFA	MP6-XT22	Biolegend
IL1B	NJTEN3	eBioscience
IL6	MP5-20F3	eBioscience
